# Literary Notes

**Published:** 1907-02

**Authors:** 


### LITERARY NOTES.

P. Blakiston, Son and Company, Philadelphia, announce that timeliness of interest, aside from any other condition, lends especial importance to the early publication of Foods and Their Adulteration, by Harvey W. Wiley, M.D., to be immediately followed by a companion volume, Beverages and Their Adulterations. Dr. Wiley is Chief Chemist to the United States Department of Agriculture, at Washington, and his wide researches in the interests of purity in food commodities give anything he might write on the subject an authoritativeness that is unquestioned. The fact that the new National Food and Drugs Law became effective January, 1907, and that public interest in it is now intense, will no doubt result in a demand for both volumes. The books will be generously illustrated from original photographs and drawings.

---

W. B. Saunders Company, Philadelphia and London, have just issued a revision of their illustrated catalogue of medical, surgical and scientific publications. This is one of the most elaborate and useful catalogues we have seen. The descriptions of the books are full, the specimen illustrations representative of the pictorial feature of the books from which they are taken, and the mechanical get-up is entirely in keeping with the high order of the context. The authors listed are men of eminence in every branch of medical science. The catalogue is well worth having, and a copy will be sent free upon request.

